# Alternative splicing profiling provides insights into the molecular mechanisms of peanut peg development

**DOI:** 10.1186/s12870-020-02702-y

**Published:** 2020-10-23

**Authors:** Xiaobo Zhao, Chunjuan Li, Hao Zhang, Caixia Yan, Quanxi Sun, Juan Wang, Cuiling Yuan, Shihua Shan

**Affiliations:** Shandong Peanut Research Institute, Qingdao, China

**Keywords:** *Arachis hypogaea*, Alternative splicing, Full-length transcriptome, Peg development, Gravitropism

## Abstract

**Background:**

The cultivated peanut (*Arachis hypogaea*) is one of the most important oilseed crops worldwide, and the generation of pegs and formation of subterranean pods are essential processes in peanut reproductive development. However, little information has been reported about alternative splicing (AS) in peanut peg formation and development.

**Results:**

Herein, we presented a comprehensive full-length (FL) transcriptome profiling of AS isoforms during peanut peg and early pod development. We identified 1448, 1102, 832, and 902 specific spliced transcripts in aerial pegs, subterranean pegs, subterranean unswollen pegs, and early swelling pods, respectively. A total of 184 spliced transcripts related to gravity stimulation, light and mechanical response, hormone mediated signaling pathways, and calcium-dependent proteins were identified as possibly involved in peanut peg development. For aerial pegs, spliced transcripts we got were mainly involved in gravity stimulation and cell wall morphogenetic processes. The genes undergoing AS in subterranean peg were possibly involved in gravity stimulation, cell wall morphogenetic processes, and abiotic response. For subterranean unswollen pegs, spliced transcripts were predominantly related to the embryo development and root formation. The genes undergoing splice in early swelling pods were mainly related to ovule development, root hair cells enlargement, root apex division, and seed germination.

**Conclusion:**

This study provides evidence that multiple genes are related to gravity stimulation, light and mechanical response, hormone mediated signaling pathways, and calcium-dependent proteins undergoing AS express development-specific spliced isoforms or exhibit an obvious isoform switch during the peanut peg development. AS isoforms in subterranean pegs and pods provides valuable sources to further understand post-transcriptional regulatory mechanisms of AS in the generation of pegs and formation of subterranean pods.

**Supplementary information:**

**Supplementary information** accompanies this paper at 10.1186/s12870-020-02702-y.

## Background

Peanut (*Arachis hypogaea* L.) is a widely cultivated oil and cash crop in the world. As one of the members of the Fabaceae family, however, peanut is a unique plant distinct from other legumes, which has the features of the aerial cleistogamous flowers and the subterranean pods [1]. After overground double fertilization, the gynophore develops a specialized geotropic aerial peg by elongation of meristematic cells locating in basal of ovary. The aerial peg grows towards the ground by gravity and penetrates into soil to develop the subterranean pods. The subterranean pod then develops embryo and produces seed underground [2]. The successful generation of pegs and formation of subterranean pods play crucial roles in the production of peanut [3]. Therefore, it is valuable to study the peanut pod to fully understand the mechanisms of peanut reproductive development. To date, many studies have been performed on the peanut pod to explore the mechanisms of its formation and development using molecular approaches [4–10]. The research presented that a large number of genes and proteins were involved in multiple biological processes during the peg development, such as gravitropic repossess, light and medical stimulus, calcium signaling, and hormone biosynthesis and transport. Furthermore, it has been evidenced that some post-transcriptional gene regulators, such as miRNAs, were involved in the regulation of peanut peg development [11–13], suggesting that post-transcriptional regulation plays an important role in controlling peanut pod formation and development. As one of the post-transcriptional regulation mechanisms, however, little information has been reported about alternative splicing (AS) in the peanut peg formation and development.

AS, a crucial post-transcriptional regulatory mode, allows a precursor mRNA to produce multiple mRNAs by selecting different splicing sites [14]. In plants, more than 60% of genes undergo AS and most of the spliced variants have unknown functions [15]. Numerous surveys in plants have been performed on dissecting AS patterns across multiple tissues and development stages, identifying multiple novel tissues- or stage-specific isoforms and stage-dependent isoform switch for many relevant genes [16–19]. It is worth noting that the genes encoding AS transcripts do not necessarily expressed remarkably during the developmental transition, indicating that AS contribution of transcriptome is independent of transcriptional regulation [20]. The similar phenomenon is also found in large-scales transcriptome studies of gene expression and AS changes during the early stages of plant development [17, 21–23]. Therefore, the identification of preponderant AS isoform switches and of development-specific AS transcripts will provide more insights into the important post-transcriptional regulatory mechanisms in controlling early plant development. As one of the ubiquitous post-transcriptional gene regulation mechanisms, however, whether peanut development-specific AS isoforms and AS switches also play important roles in controlling peanut peg formation and development remains largely to be elucidated.

Here, we presented a comprehensive full-length (FL) transcriptome profiling of AS isoforms during the peanut peg and early pod development, providing evidence that many genes are related to gravity stimulation, light and mechanical response, hormone mediated signaling pathways, and calcium-dependent proteins undergoing AS express development-specific spliced isoforms or exhibit an obvious isoform switch. The identification of specific AS isoforms in subterranean pegs and pods provides valuable sources to further understand post-transcriptional regulatory mechanisms of AS in the generation of pegs and formation of subterranean pods.

## Results

### Peanut transcriptome sequencing

To reconstruct a comprehensive FL transcriptome landscape in peanut, eight barcoded SMRT Bell libraries were constructed from tissues of roots, leaves, shoot tips, flowers, peg samples of S1 (aerial peg tips), S2 (subterranean peg tips), S3 (subterranean unswollen peg tips) and S4 (ubterranean swelling pod) (NCBI Accession Number: PRJNA643877), resulting in 394,047,898 raw subreads, with an average of 49,255,987 subreads per sample, of which more than 95% were lower than 5 kbp in read length (Table S1). After self-correction among subreads, a total of 3,649,775 high-quality reads of insert were generated. Of these reads, based on presence of 3′-primers, 5′-primers, and poly(A) tails, 643,565 (17.63%) were grouped into nFL (non full-length) reads and 3,006,210 (82.37%) were grouped into FL reads (Table 1). By removing chimeric transcripts, final 2,814,161 (77.11%) FLNC (full-length non-chimeric) reads were obtained employed for subsequent analysis.

**Table 1:** Summary of high-quality reads of insert

Category	Shoot tip	Root	S1	S2	S3	S4	Flower	Leave	Total
Reads of insert	400,282	396,926	498,928	488,048	362,137	381,966	682,097	439,391	3,649,775
Five primer reads	333,260	334,611	442,427	421,034	309,595	319,060	576,311	366,319	3,102,617
Three primer reads	358,826	359,638	462,327	444,666	327,281	349,521	624,277	401,506	3,328,042
Poly(A) reads	273,138	295,784	411,998	389,290	286,974	288,584	523,061	332,753	2,801,582
nFL reads	79,790	73,124	67,968	77,246	59,857	73,324	126,901	85,355	643,565
FL reads	320,492	323,802	430,960	410,802	302,280	308,642	555,196	354,036	3,006,210
FLNC reads	274,113	296,760	413,600	390,508	287,813	290,397	525,991	334,979	2,814,161
Chimeric reads	46,379	27,042	17,360	20,294	14,467	18,245	29,205	19,057	192,049
Average length of FLNC reads	743.66	912.62	1095.86	1018.82	1009.25	995.44	1355.51	1143.87	1034.378

*nFL reads* non-full-length reads, *FL* full-length reads, *FLNC reads* Full-length non-chimeric reads

### Reconstruction of peanut full-length transcripts in peanut

**Fig. 1:**
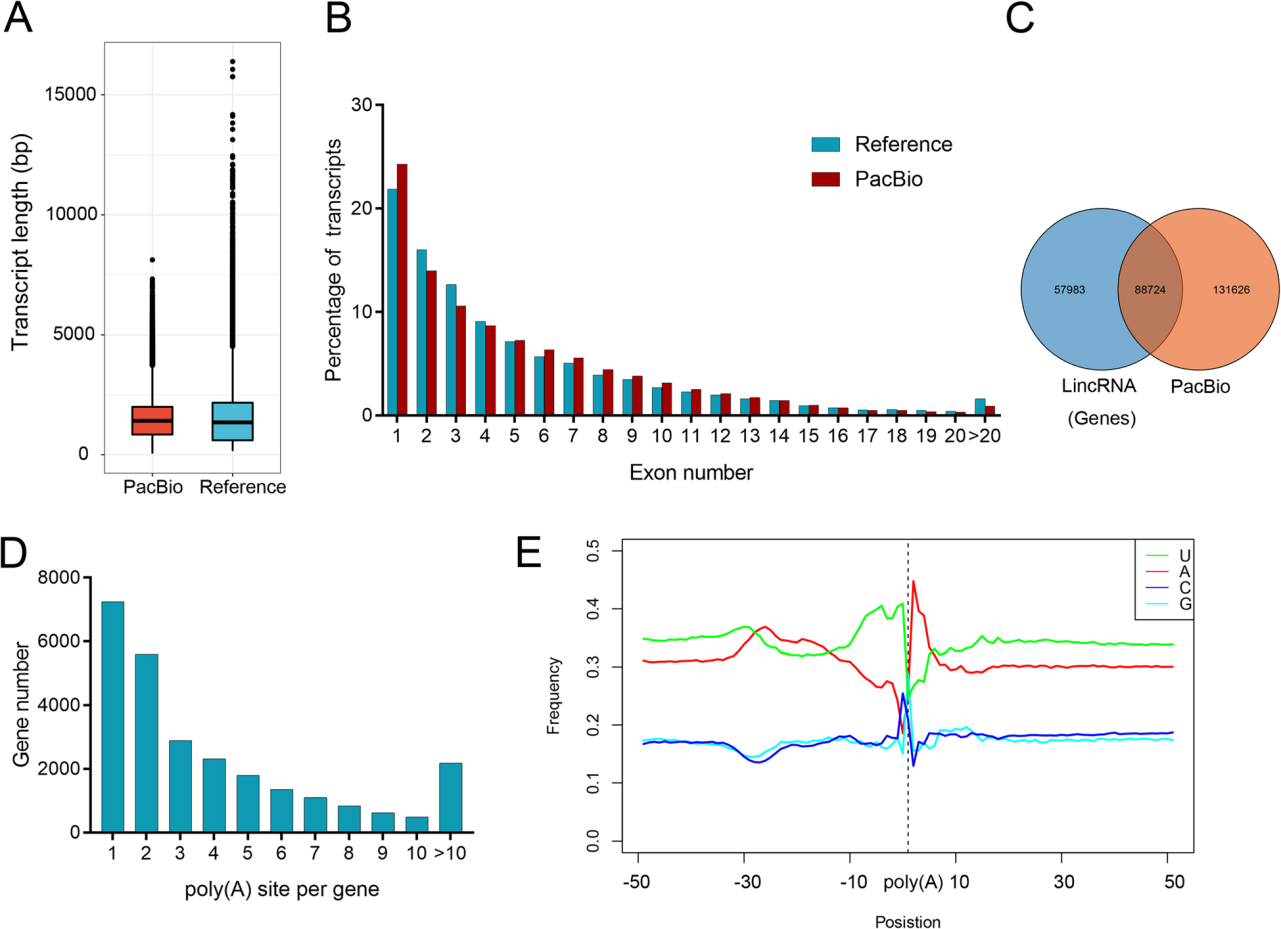
Characterization of peanut transcriptome using Iso-Seq. **a** Box-plots show distribution patterns of transcript length in reference and PacBio Iso-Seq data. **b** Distribution of exon number per isoform in reference and PacBio Iso-Seq data. **c** Venn diagram shows the number of full-length long intergenic noncoding RNAs (lincRNAs) detected in PacBio Iso-Seq data. **d** Distribution of the number of poly(A) sites per gene. **e** Relative frequency of each nucleotide around poly(A) cleavage sites. Sequences in the upstream (−50 bp) and downstream (+ 50 bp) of each poly(A) cleavage site were analyzed

To obtain nonredundant transcripts, the FLNC reads generated were used for clustering analysis (Fig. S1a). First, using GMAP, 2,801,582 (99.55%) FLNC reads were mapped to peanut reference genome, of which 703,199 (25.10%) were mapped and 2,098,383 (74.90%) were uniquely mapped (Fig. S1b). The mapped FLNC reads were then grouped into consensus transcripts, resulting in 247,885 unique FL transcripts originating from 53,618 gene models (Fig. S2). The average length of these transcripts (1475 bp) was relatively shorter than those of peanut reference transcripts (1571 bp) (Fig. 1a). Of these FL transcripts obtained, 86,318 transcripts possessed exon, of which 67,457 (78.15%) transcripts had at least two exons (Fig. 1b).

Among of the FL transcripts obtained, 223,855 transcripts were mapped to 46,388 known peanut reference gene models. By comparing with reference gene annotations, 28,845 known gene models corresponded to 53,553 FL transcripts were extended in 3′ or 5′ boundaries. To update gene structures, ORFs (open reading frames) and untranslated regions (UTRs) of FL transcripts were predicted via ANGEL pipeline and optimized parameters trained by known gene models of diploid and tetraploids peanut, a total of 28,686 gene models were finally updated. Meanwhile, by applying cDNA_Cupcake program, a total of 63,070 putative fusion gene models involving 63,091 fusion transcripts were identified, in which most of fusion transcripts were detected from fusion events of interchromosomal (Fig. S2).

By dissecting the FL transcripts aligned to peanut reference genome, a total of 24,030 novel alternative splicing transcripts located in the intergenic regions were identified from 7230 gene models. Of these transcripts, 10,062 (4490 genomic loci) (Fig. S2) were defined as long intergenic noncoding RNAs (lincRNAs), based on two predictors of CPAT and PLEK. The most of these lincRNAs possessed at least two exons, with length distribution ranging from 0.2 to 5.0 kbp (Fig. S1c, d). Among of these putative lincRNAs, a total of 131,626 lincRNAs were identified as novel lincRNAs by comparing with our previous data set (Fig. 1c).

Analysis of the 3′ ends of FL transcripts helped us to identify alternative polyadenylation sites in peanut. Of the 53,618 gene models detected, 47,915 genes possessed at least one poly(A) site, and 11,109 genes had at least 5 poly(A) sites, with an average number of 2.23 poly(A) sites per genes (Fig. 1d). By dissecting the flanking nucleotide sequence features of all poly(A) sites, an obvious nucleotide bias was observed with uracil (U) enrichment upstream and adenine (A) downstream around the poly(A) sites in 3′ UTRs. Two motifs of AAUAAA and UGUA were found to be the common features located in the upstream of the poly(A) sites (Fig. 1e).

### Identification of alternative splicing events

**Fig. 2:**
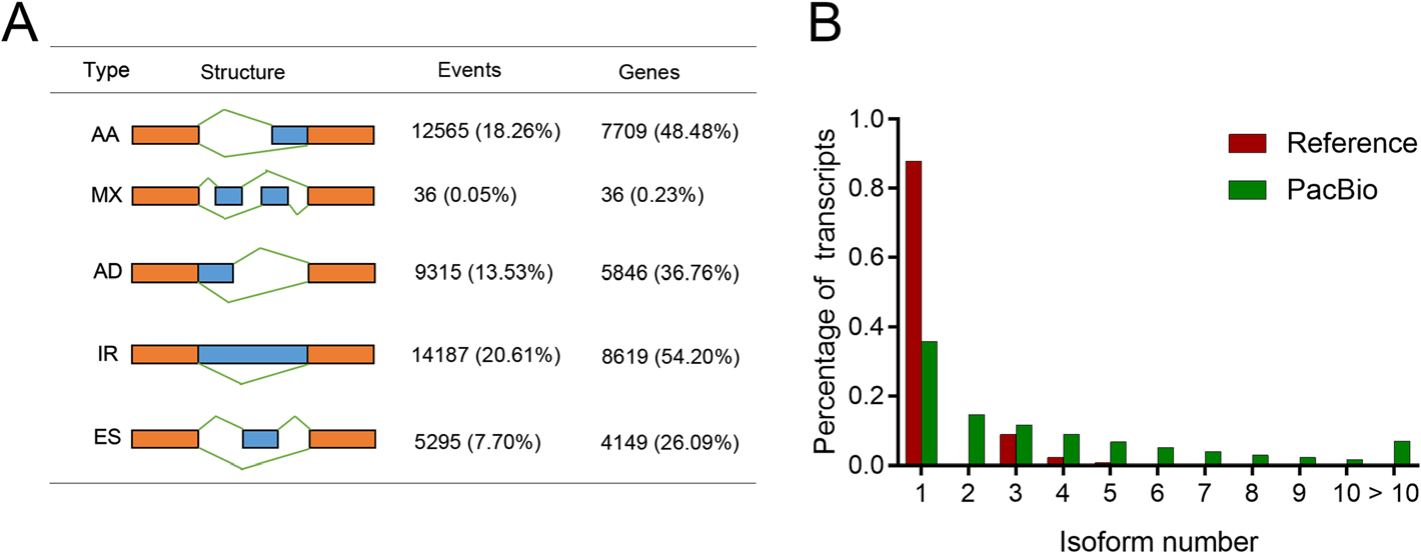
AS analysis of peanut with Iso-Seq reads. **a** Classification of AS events. Cartoons show AS events: alternative adaptor (AA), mutually exclusive exon (MX), alternative donor (AD), intron retention (IR), and exon skipping (ES). The number and percentage of AS events and associated genes are shown. **b** Distribution of the number of isoforms per gene. The red and green represent the numbers of isoforms for all genes in reference and PacBio data, respectively

The FL transcripts obtained and AStalavista program were used to identify the alternative splicing events in peanut. As a result, a total of 68,823 AS events were detected from 15,903 genes (Table S2). Of these AS events detected, a total of 41,398 (60.15%) were well classified into 5 major AS events, including IR (intron retention), AA (alternative adaptor), AD (alternative donor), ES (exon skipping), and MX (mutually exclusive exon). As one of the most abundant AS events, IR was found to possess 14,187 (20.61%) events representing 8619 (54.20%) of genes undergoing AS (Fig. 2a). AA was found to be the second most abundant having 12,565 (18.26%) events which represent 7709 (48.48%) of AS genes, followed by AD (9315, 13.53%) and ES (5295, 7.7%).

As a consequence of AS events, a total of 170,302 new FL transcript isoforms from 46,388 reference gene models were identified. Of these gene models, 5648 (12.18%) gene models within reference annotation were found to contain more than one transcript, whereas 29,837 (64.32%) in Iso-seq data were found to contain at least two transcript isoforms (Fig. 2b). By comparing with known reference transcripts, the percentage of genes containing more than five transcripts in this analysis (29.2%) was remarkably higher than that (1.0%) lying in reference genome annotation. On average, this AS events analysis detected 4.83 transcripts per gene having more 4.2-fold higher than that in the originated reference annotation (Fig. 2b).

**Fig. 3:**
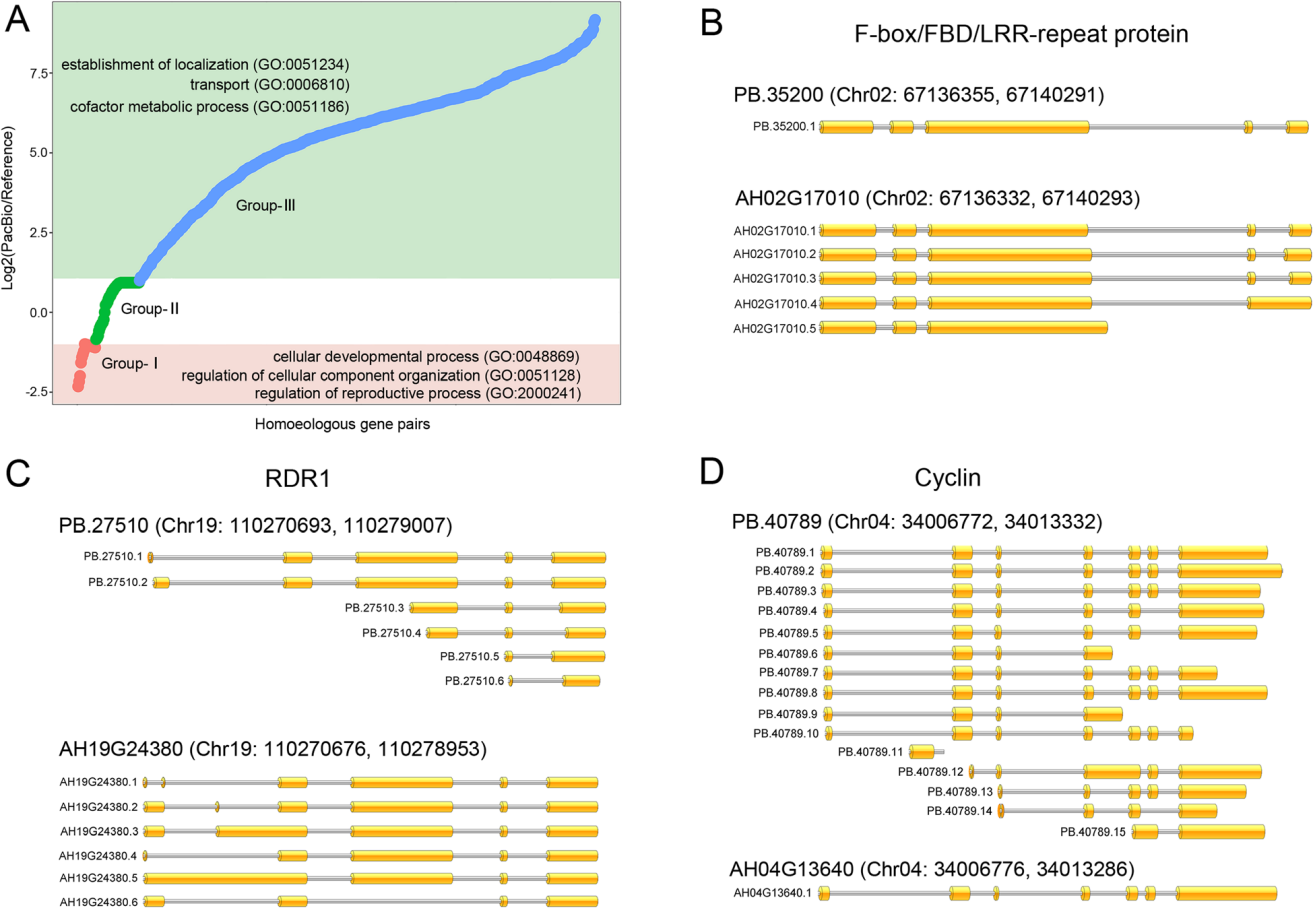
The number of alternative spliced isoforms detected in reference genome and Iso-seq data. **a** Log2 ratio of isoform numbers of alternative spliced genes in the reference genome and Iso-seq data. All of these genes were categorized into three groups: group-I (log2Ratio ≤ 1), group-II (log2Ratio > 1 and log2Ratio < 1) and group-III (log2Ratio ≥ 1). Significant GO terms are shown for genes in the group-I and group-III. **b** Transcript isoforms of alternative spliced gene F-box/FBD/LRR-repeat protein in reference genome and Iso-seq data. **c** Transcript isoforms of alternative spliced gene RDR1 (RNA-dependent RNA polymerase 1) in the reference genome and Iso-seq data. **d** Transcript isoforms of alternative spliced gene cyclin in the reference genome and Iso-seq data. In b-d, upper tracks show isoforms for genes in the Iso-seq data and lower tracks show isoforms for genes in reference genome. Yellow boxes show exons in each transcript model. Gene names in the reference genome

To compare the alternative spliced isoforms detected in reference genome and Iso-seq data, the ratio of isoforms originating from same genes between Iso-seq and reference transcripts were calculated. The results showed that 344 genes (group 1) had fewer isoforms in Iso-seq than reference data sets; 3706 genes (group 2) shared equal isoform numbers in Iso-seq and reference data sets; 24,803 genes (group 3) had relatively more isoform numbers in Iso-seq than reference data sets (Fig. 3a). GO enrichment analysis based on all transcripts obtained showed that the genes in group 1 were mainly involved in cellular developmental process, regulation of cellular component organization, and reproductive process. The genes in group 3 were enriched in establishment of localization, transport, and cofactor metabolic process. For instance, one gene *PB.35200* from group 1, encoding a F-box/FBD/LRR-repeat protein, was found containing only one isoform in Iso-seq data set, whereas in reference data sets was detected containing 5 isoforms (Fig. 3b). Most of these isoforms detected in group 1 were transcribed by the AS events of AA and AD. A gene *PB.27510* from group 2, encoding RNA-dependent RNA polymerase 1, was shown to transcribe 6 transcripts in both Iso-seq and reference data sets (Fig. 3c). One gene *PB.40789* from group 3, encoding a cyclin protein, was detected to produce 15 isoforms in Iso-seq data set, whereas in reference data sets was shown to transcribe only one transcript isoform (Fig. 3d). These results further suggested that AS events contributed to the transcriptome complexity in peanut.

### Tissue-specific alternatively spliced isoforms

**Fig. 4:**
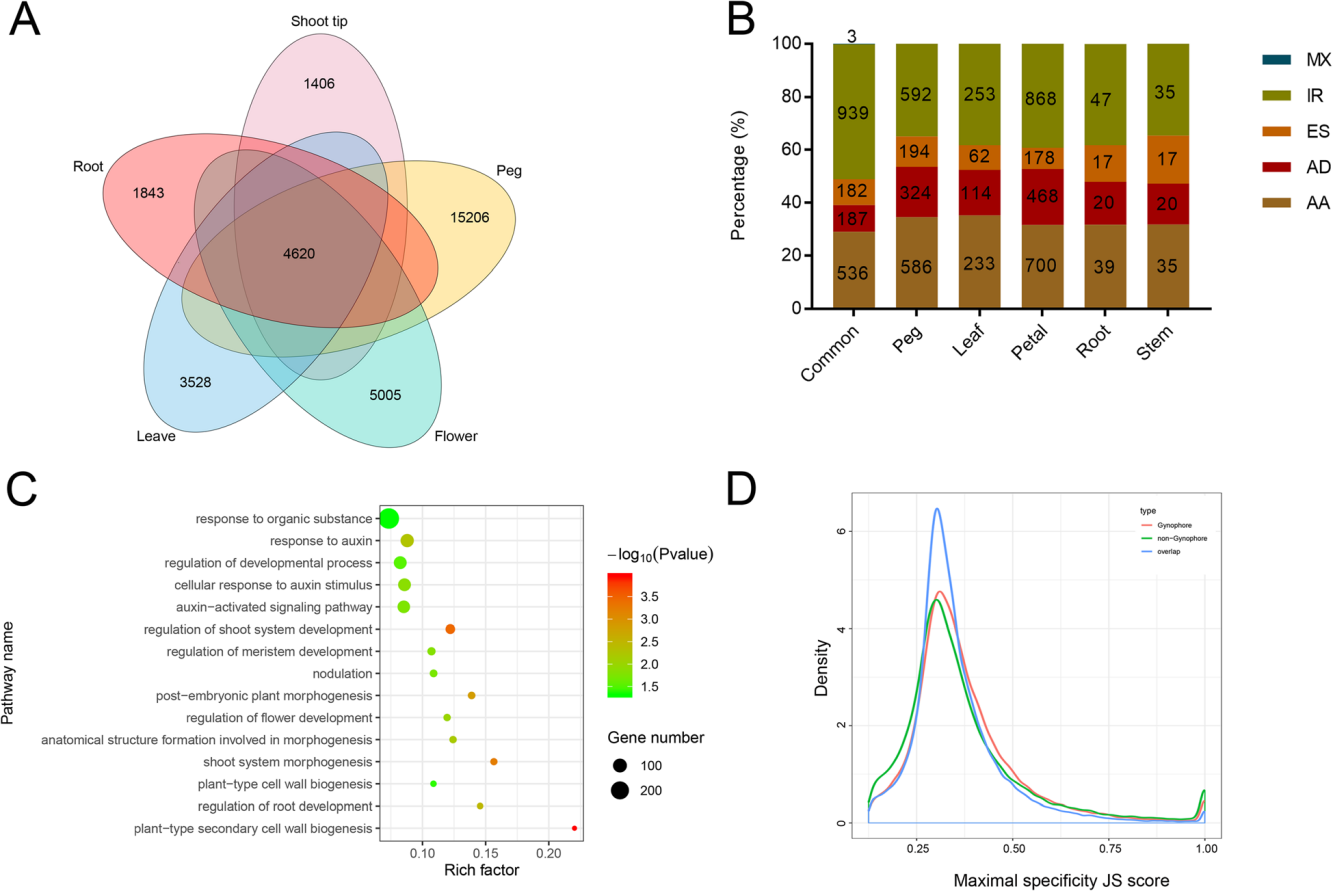
Identification of tissue-specific alternatively spliced isoforms. **a** Venn diagram shows numbers of tissue specific alternatively spliced isoforms. **b** Percentage of five AS types of specific alternatively spliced isoforms in each tissue. Common represents the share isoforms of five tissues. **c** GO analysis of specific alternatively spliced isoforms in peg tissue. The top 15 GO terms are shown. The Y-axis on the left represents GO terms, and the X-axis indicates the “Rich factor” represented by the ratio of specific isoform numbers to total annotated isoform numbers of each term. The area of a circle represents specific isoform number. The lower the *p*-value, the more significant the enrichment. **d** The distributions of maximal tissue specificity scores (Jensen–Shannon, JS scores) for isoforms in the three categories

To identify tissue-specific isoforms, the FL transcript isoforms from all tissues were compared. Venn diagram showed that 1843, 3528, 1406, 5005, and 15,206 isoforms were uniquely detected in roots, leaves, shoot tips, flowers, and peg tissues, respectively (Fig. 4a). A total of 4620 overlapped alternative spliced isoforms were identified among all tissues (Fig. 4a). The most of these isoforms were produced from AS events of IR, AA, and AD (Fig. 4b). For peg tissues, 11,112 of 15,206 specific isoforms were transcribed from 5467 genes by AS events. GO enrichment analysis showed that these isoforms were mainly involved in multiple metabolic processes, biosynthetic process, protein modification process, signal transduction, cellular stress responses, such as response to organic substance (auxin, organonitrogen compound) and nitrogen compound, and plant growth and development regulation process, such as nodulation, tissues development, and morphogenesis (Fig. 4c).

To further evaluate the reliability of tissue-specific isoforms, the tissue specificity score of each isoform was calculated based on the isoform expression levels obtained by high through-put Illumina sequencing data. Results showed that the distribution of JS scores of peg tissue and non-peg tissue specific isoforms were remarkably higher than that overlapped isoforms (Kolmogorov-Smirnov test, *P* < 2.2 × 10–16) (Fig. 4d). The result indicated that the tissue-specific isoforms detected by Iso-seq had a high consistence with their expression patterns by high through-put Illumina sequencing data, reflecting the reliability of these tissue-specific isoforms detection.

### Expression dynamic of isoforms across peg developmental stages

**Fig. 5:**
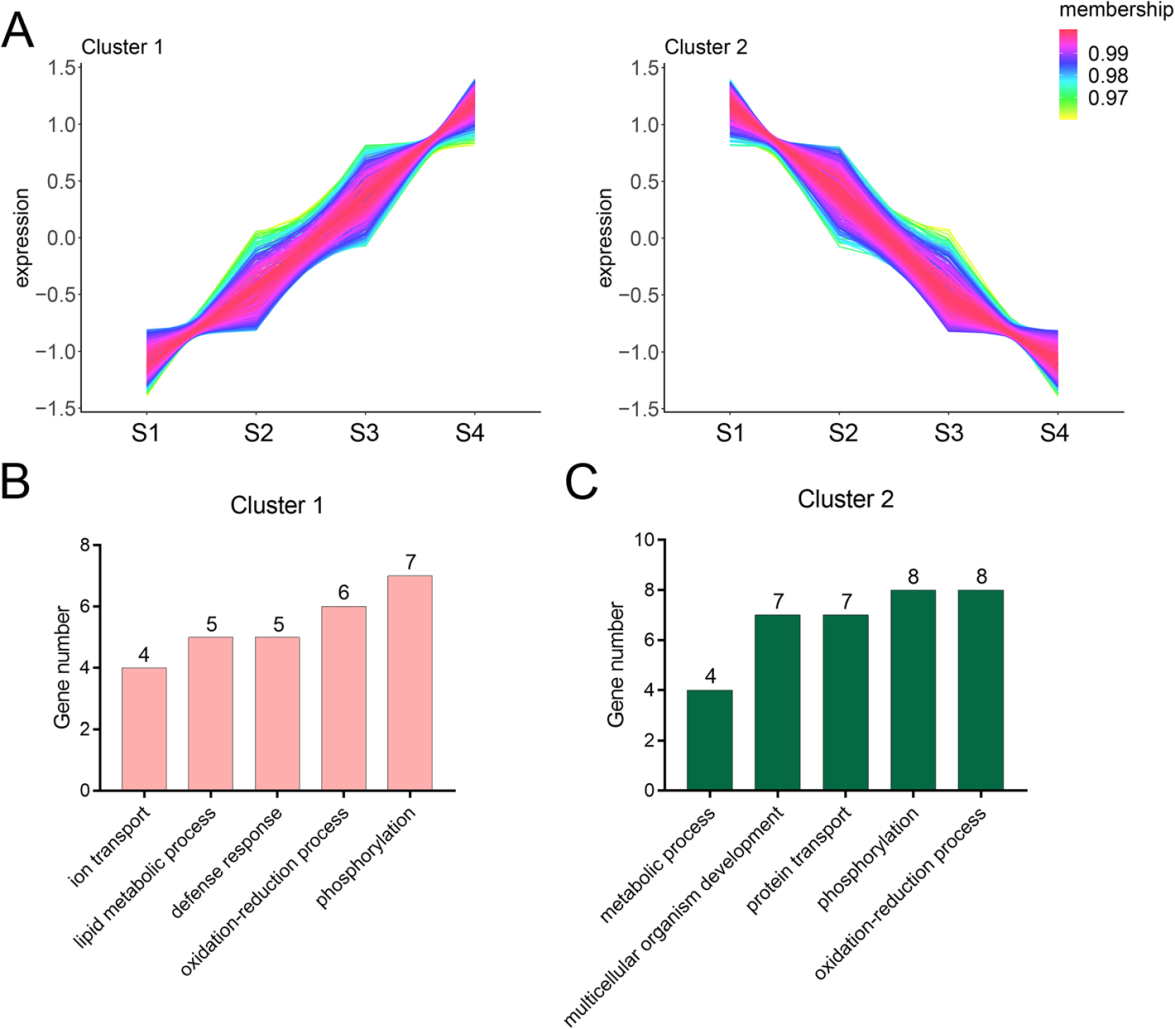
Expression dynamic of differentially expressed isoforms across peg developmental stages. **a** Cluster analysis of differentially expressed isoforms at different developmental stages by performing TCseq software. **b** Histogram shows the top 5 GO terms in cluster1. **c** Histogram shows the top 5 GO terms in cluster 2

To investigate the role of alternatively spliced isoforms on peg development, the expression levels of peg tissue-specific alternatively spliced isoforms were compared among four peg developmental stages. A total of 690 alternatively spliced isoforms were found differentially expressed at four peg developmental stages. By using TCseq software, these isoforms were further divided into 2 distinct clusters. (Fig. 5a). Cluster 1 contained 340 isoforms, the expression of which consistently increased at four peg developmental stages. GO function analyses were performed on these isoforms and many functional terms were enriched including phosphorylation, oxidation-reduction process, lipid metabolic process, ion transport, and defense response. Among them, many expression levels of isoforms encoding transcription factors were increased at four peg developmental stages, including members of heat stress, Myb, GATA, WRKY, bHLH140, Trihelix, and other families (Fig. 5b and Table S3). Furthermore, four isoforms encoding splicing factor were found constitutively expressed across four peg developmental stages, including splicing factor 3B and serine/arginine-rich splicing factor members, suggestion that the splicing factors increased the complexity of transcriptional regulation during peg development.

Contrary to the expression pattern of cluster 1, cluster 2 contained 350 isoforms, of which the expression levels of the isoforms exhibited consistently decreased at four peg developmental stages. GO functional enrichment analysis showed that the isoforms were mainly involved in multicellular organism development, protein transport, and metabolic process. Among of these isoforms, many encoding transporter family members exhibited decreasingly expressed during peg development, including ABC transporters (ABC transporter family B, C, F, and G), boron transporter, plastidic glucose transporter, and bidirectional sugar transporter (Fig. 5c and Table S3). Four isoforms encoding auxin efflux carriers (PB.13251.3 and PB.14190.1) and auxin-induced proteins (PB.34545.3 and PB.51026.1) were found constitutively decreased across four peg developmental stages. The isoforms encoding tubulin (PB.17695.3) and early nodulin-like protein (PB.9298.3) showed decreased expression patterns during peg development stages. Furthermore, the isoforms encoding cellulose synthase exhibited decreasingly expressed patterns across developmental stages, suggesting the reduced synthesis of cellulose through whole ped developmental stages.

### Stage-specific splicing isoforms

**Fig. 6:**
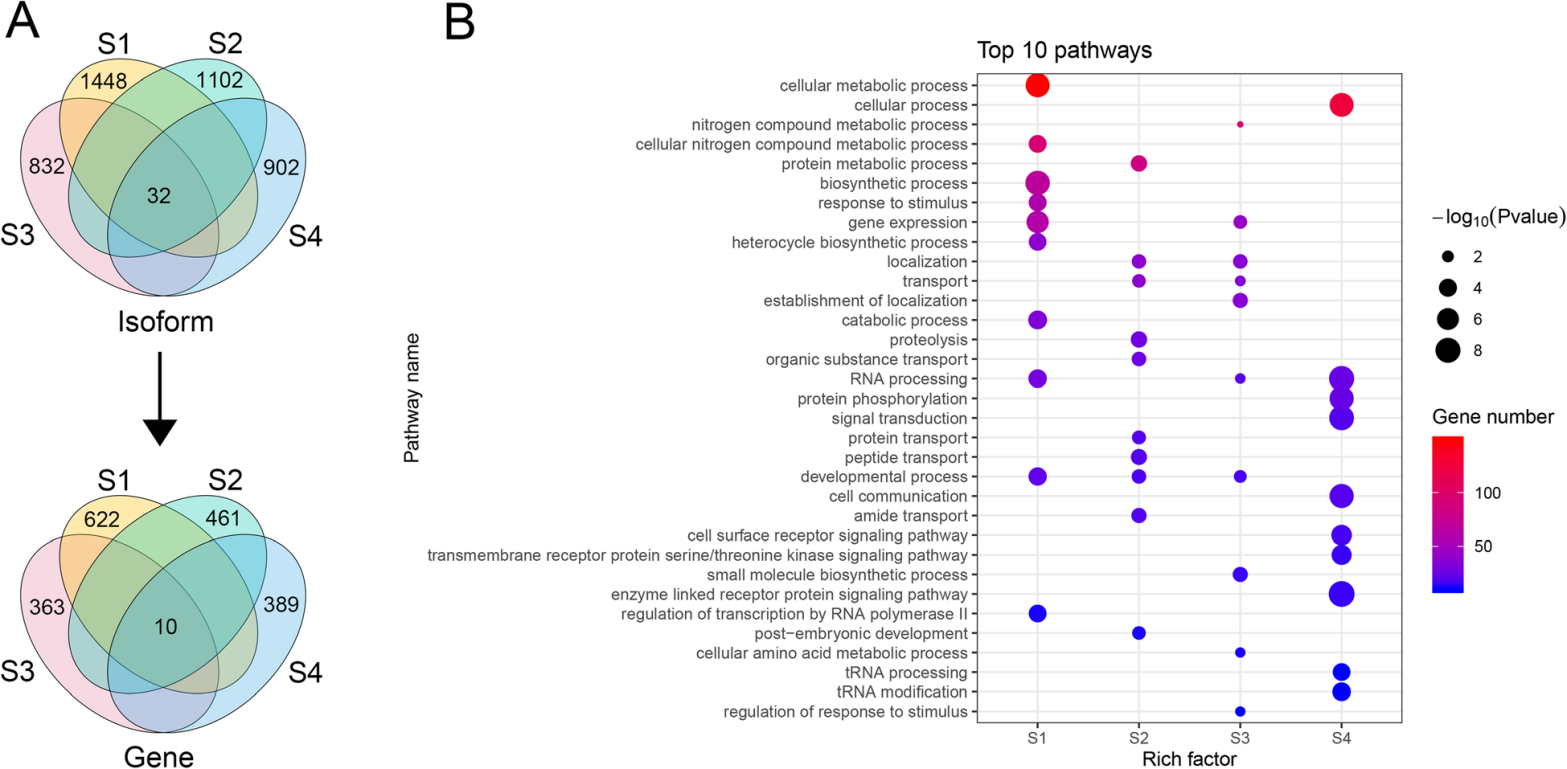
Characterization of stage-specific splicing isoforms. **a** Venn diagrams show the distribution of AS isoforms and genes in different stages of peg development. **b** GO term analysis of stage-specific isoforms and the top 10 GO terms (rank by p-value) in each stage are shown. The Y-axis on the left represents GO terms, and the X-axis indicates the “Rich factor” represented by the ratio of specific isoform numbers to total annotated isoform numbers of each term. The area of a circle represents specific isoform number. The lower the *p*-value, the more significant the enrichment

To elucidate the role of stage-specific splicing isoforms in peanut peg development, the exclusive isoform expression levels in peanut were compared across peg tissues at four developmental stages. The result showed that 1448, 1102, 832, and 902 uniquely expressed isoforms were alternatively spliced from 622, 461, 363, and 389 genes at stage of S1, S2, S3, and S4, respectively (Fig. 6a). GO functional enrichment analysis of these stage alternatively spliced isoforms showed that the process of metabolic, biosynthetic, catabolic, and developmental, response to stimulus, and RNA processing were mainly enriched in S1; nitrogen compound or protein metabolic process, protein or organic substance transport, proteolysis, cellular, macromolecule, or protein localization, biological process regulation, and post-embryonic development were the major functions enriched in S2; S3 enriched in the process of nitrogen compound or cellular amino acid metabolic, small molecule biosynthetic process, localization, transport, and developmental process; the specific alternatively spliced isoforms in S4 mainly participated in cellular process, protein phosphorylation, signal transduction, and cell communication (Fig. 6b and Table S4).

**Fig. 7:**
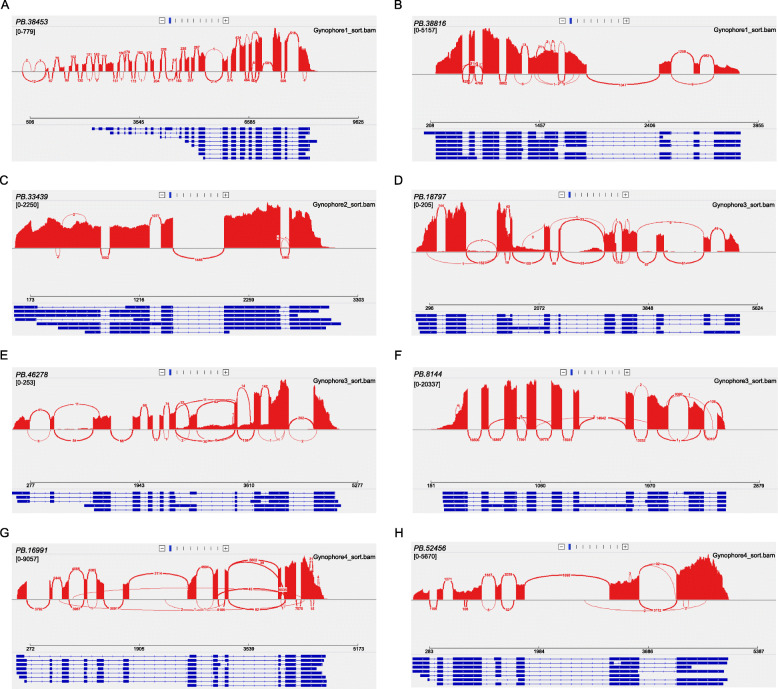
Sashimi plot shows transcript isoforms of PB.38453 (**a**), PB.38816 (**b**), PB.33439 (**c**), PB.18797 (d), PB.46278 (**e**), PB.8144 (**f**) PB.16991 (**g**), and PB.52456 (**h**). Peaks in red represent short-read coverage. For each isoform, blocks in blue represent exons, and lines between blocks represent introns

Among the 622 genes with specifically spliced isoforms at S1 stage, *PB.38453*, encoding a puromycin-sensitive aminopeptidase, had the largest number of alternatively spliced isoforms due to IR and AD events, of which 2 isoforms were from an IR event and 6 isoforms were from an AD event (Fig. 7a). The second gene, *PB.38816*, encoding an inositol-3-phosphate synthase, produced 7 alternatively spliced isoforms via IR and AA event, of which were specifically expressed at S1 stage with expression levels distribution from 0.15 to 65 (Fig. 7b). The gene *PB.33439*, which was responsible for encoding a primary amine oxidase, had the greatest number of specifically expressed isoforms at S2 stage. The gene was specifically spliced into 7 isoforms with expression levels ranging from 0.1 to 35 by two AD events (Fig. 7c). Similar phenomenon was observed at S3 stage for gene *PB.18797* encoding for a high affinity nitrate transporter, *PB.46278* coding for an LRR receptor-like serine/threonine-protein kinase, and a gene *PB.8144* encoding for a l-ascorbate peroxidase. All three genes had 5 alternatively spliced isoforms resulting from AD and IR events (Fig. 7d-f). A gene *PB.16991*, encoding a glutamine synthetase nodule isozyme, was specifically spliced into 8 isoforms by an AD and AA event (Fig. 7g), of which were specifically expressed at S4 stage with expression levels ranging from 0.12 to 65.5. Similarly, a gene *PB.52456*, coding for SIEVE ELEMENT OCCLUSION B protein, was observed to have 7 alternatively spliced isoforms at S4 stage due to an AD and IR event (Fig. 7h). All these alternatively spliced isoforms were specifically expressed at S4 stage with different expression levels. Furthermore, these results suggested that the stage-specific splicing isoforms coming from AS events may play an important role in peanut peg development.

## Discussion

A comprehensive FL transcriptome atlas of peanut were reconstructed from tissues of roots, leaves, shoot tips, flowers, and peg tissues by PacBio Iso-seq. Using Iso-seq data, we detected multiple AS events associated with peg tissue and developmental stage specific FL transcripts. We identified 1448, 1102, 832, and 902 alternatively spliced transcripts in peanut S1, S2, S3, and S4, respectively, including 184 alternatively spliced transcripts related to light and mechanical response, gravity stimulation, hormone mediated signaling pathways, and calcium-dependent proteins, which were considered playing important roles in peanut peg development, such as peg elongation and early pod formation.

S1 pegs represented the fertilized aerial pegs which sensed gravity and bended downward. In this stage, we identified multiple specifically expressed transcripts related to light and mechanical stimulus, gravity stimulation, hormone response factors, and calcium-dependent protein kinases and sensing receptors (Table S5). Among these transcripts, we found 21 transcripts related to light and mechanical stimulus, including vacuolar protein sorting-associated protein (9 transcripts), peroxidase-related proteins (6 transcripts), V-type proton ATPase subunit (4 transcripts), and vacuolar cation/proton exchanger 5 (2 transcripts), which were reported to play key roles in peanut peg elongation and pod development [4, 7, 9, 24]. Previous studies showed that ABC transporters, microtubules, microtubule-associated proteins, and heat shock proteins play important roles in responding to plant gravity stimulation [25–27]. In this study, we identified 20 specifically expressed transcripts related to gravity stimulation, including 8 ABC transporter transcripts, 5 alpha-tubulin transcripts, 3 microtubule-associated protein transcripts, two microtubule-binding protein TANGLED transcripts, and two small heat shock protein transcripts. Notably, two gravitropic genes, *ALTERED RESPONSE TO GRAVITY* (ARG1) and *GRAVITROPISM DEFECTIVE 2* (GRV2), which were well characterized to affect plant growth in response to gravity [28–30], were detected each alternatively transcribed 2 isoforms and uniquely expressed only in S1 pegs. It was reported gravity perception could give rise to asymmetric redistribution of auxin and make the plant organs bending away or growing towards gravity vector [31, 32]. We identified 15 transcripts related to hormone response factors, including 7 auxin response factors, 2 auxin-responsive protein IAA26, 2 ethylene-overproduction protein 1, 2 ethylene-responsive transcription factor RAP2–12, and 2 IAA-amino acid hydrolase ILR1-like 6, which were specifically expressed in S1 pegs. Gravity stimulation response could increase Ca^2+^ level of cytoplasmic and further promote cell wall extensibility, which changed the polar transport of auxin [33, 34]. We found 6 specifically expressed transcripts related to calcium-dependent protein kinases and sensing receptors, including calcium sensing receptor (3 transcripts), and calcium-dependent protein kinase (3 transcripts). For instance, we identified 14 cell wall structure-related transcripts, including 10 villins, 2 polygalacturonases, and 2 expansins. Villins were involved in regulating plant actin dynamics, such as the growth of pollen tubes and root hairs [35, 36]. Expansins play vital roles in multiple morphogenetic processes, such as plant cell wall elongation and extension, growth of pollen tubes and root hairs, regulation of auxin action [37, 38]. Our results showed that villins and expansins may contribute to the elongation of S1 pegs. Another gene, GDSL esterase/lipase, which was reported to have functions in regulating ethylene signaling components to modulate systemic immunity in *Arabidopsis* [39, 40], was identified to alternatively transcribed 2 isoforms and only expressed in S1 pegs.

S2 represented the subterranean pegs which penetrated into soil for 24 h, which exhibited different morphological and physiological characteristics comparing with S1 pegs. We identified multiple alternatively spliced transcripts that were specifically expressed in S2 pegs (Table S5), of which mainly related to mechanical stimulus, gravity stimulation, auxin transporter and responsive proteins, transporters, and protein kinases. Twenty-two alternatively spliced transcripts related to gravity stimulation (10 transcripts) and mechanical stimulus (12 transcripts) were specifically expressed in S2 pegs, suggesting that the transcripts contributed to the peanut peg elongation after penetration into soil within 24 h. A gravitropic gene, *SHOOT GRAVITROPISM 6* (*SGR6*), which was reported to be involved in regulation of morphological and vacuolar membranes structures changes in gravity-sensing endodermal cells in *Arabidopsis* [41], were identified to have 3 isoforms by an AA event and specifically expressed only in S2 pegs. Further analysis identified 5 transcripts likely to be auxin transport and responsive including 2 involved in auxin transporter-like protein 4 and 3 in auxin-responsive protein IAA30. We identified multiple specifically expressed transporter transcripts in S2 pegs, including GABA transporter 1 (2 transcripts), magnesium transporter NIPA6 (4 transcripts), sulfate transporter 4.2 (4 transcripts), protein transport protein Sec24-like At3g07100 (3 transcripts), sugar transporter ERD6-like 6 (2 transcripts), tonoplast dicarboxylate transporter (3 transcripts), and tonoplast dicarboxylate transporter (2 transcripts), of which were in consistence with previous transcriptome study in peanut [4]. A large number of transcripts coding for serine/threonine protein kinase proteins were identified to be involved in cold, salt stress and in regulation of plant growth and development through autophosphorylation [42, 43].

S3 pegs, unlike S2, represented the subterranean pegs reorientation against gravity after penetration into soil for three days. We identified many alternatively spliced transcripts related to mechanical stimulus, such as peroxidases, vacuolar-sorting receptors, and V-type proton ATPases and many hormone response proteins, i.e., auxin responsive and ethylene-overproduction proteins, suggesting that these transcripts play roles in underground pegs development. Similar with S2 pegs, we found multiple transcripts related to serine/threonine protein kinase proteins, especially for LRR receptor-like serine/threonine-protein kinase (10 transcripts) which were involved in abscisic acid early signaling and acted as an essential regulator in *Arabidopsis* embryonic pattern formation and cotyledon primordia generation [44–46]. Notably, we detected many AS transcripts which were involved in regulation of development process in S3 pegs. A gene, *EMBRYO DEFECTIVE 1674* (*EMB1674*), had two isoforms due to an AA event, which was involved in regulation of normal embryo development in *Arabidopsis* [47]. The gene *BASIC PENTACYSTEINE4* (*BPC4*), having two isoforms as an AD event, functioned as a positive transcriptional regulator that was involved in developmental processes in *Arabidopsis* [48]. A gene *Aberrant root formation protein 4* (*ALF4*), encoding a nuclear-localized protein, had two isoforms due to an IR event, which play an essential role in *Arabidopsis* lateral root formation [49]. Meantime, we detected some genes involving in light regulation of development, for example, *FAR1-RELATED SEQUENCE 5* (*FRS5*), had three isoforms due to AD, AA, and ES events, which functioned as a transcription activator involving in regulation of light control of development in *Arabidopsis* [50]; *XAP5 CIRCADIAN TIMEKEEPER* (*XCT*) had two isoforms through an AA event which was involved in coordinating light signals for photomorphogenesis and circadian clock in *Arabidopsis* [51].

S4 represented subterranean pod undergoing swelling and elongation after soil penetration. We identified 902 alternatively spliced transcripts in S4 pods. Among of these transcripts, we detected multiple transcripts related to mechanical stimulus, auxin response proteins, calcium binding/transporting proteins, transports, cyclin proteins, and development process regulators. Seventeen transcripts related to mechanical stimulus (8 transcripts) and calcium binding/transporting proteins (7 transcripts) were identified specifically expressed in early swelling pods. Eight transcripts encoding auxin response factor 3/4 and auxin-responsive protein IAA9 were specifically expressed in S4 pods, which were involved in expression regulation of auxin response genes [52]. Multiple inorganic ions and small organic substances transporter transcripts exhibited uniquely expressed in S4 pods. One gene, *auxin transport protein BIG* (BIG), encoding a calossin-like protein, had two isoforms due to an AD event, which was involved in polar auxin transport during light-mediated stimuli [53]. Many AS transcripts encoding cyclin proteins were identified specifically expressed in early swelling pods. Of these cyclin proteins, *Cyclin-D4–1* (*CYCD4–1*) acted as activator of *Arabidopsis* root apex division and promoted seed germination [54]. In addition, we identified multiple specifically expressed transcripts related to development process regulators in S4 pods. Seven transcripts from gene *TOPLESS* (*TPL*) encoding transcriptional corepressor were identified uniquely expressed in early swelling pods, which were functioned as an essential factor in ovule development [55]. Notably, five transcripts from gene *ROOT HAIR DEFECTIVE 3* (*RHD*3) encoding GTP-binding protein were identified in swelling pods, which was involved in root or root hair cells enlargement [56–58].

## Conclusions

**Fig. 8:**
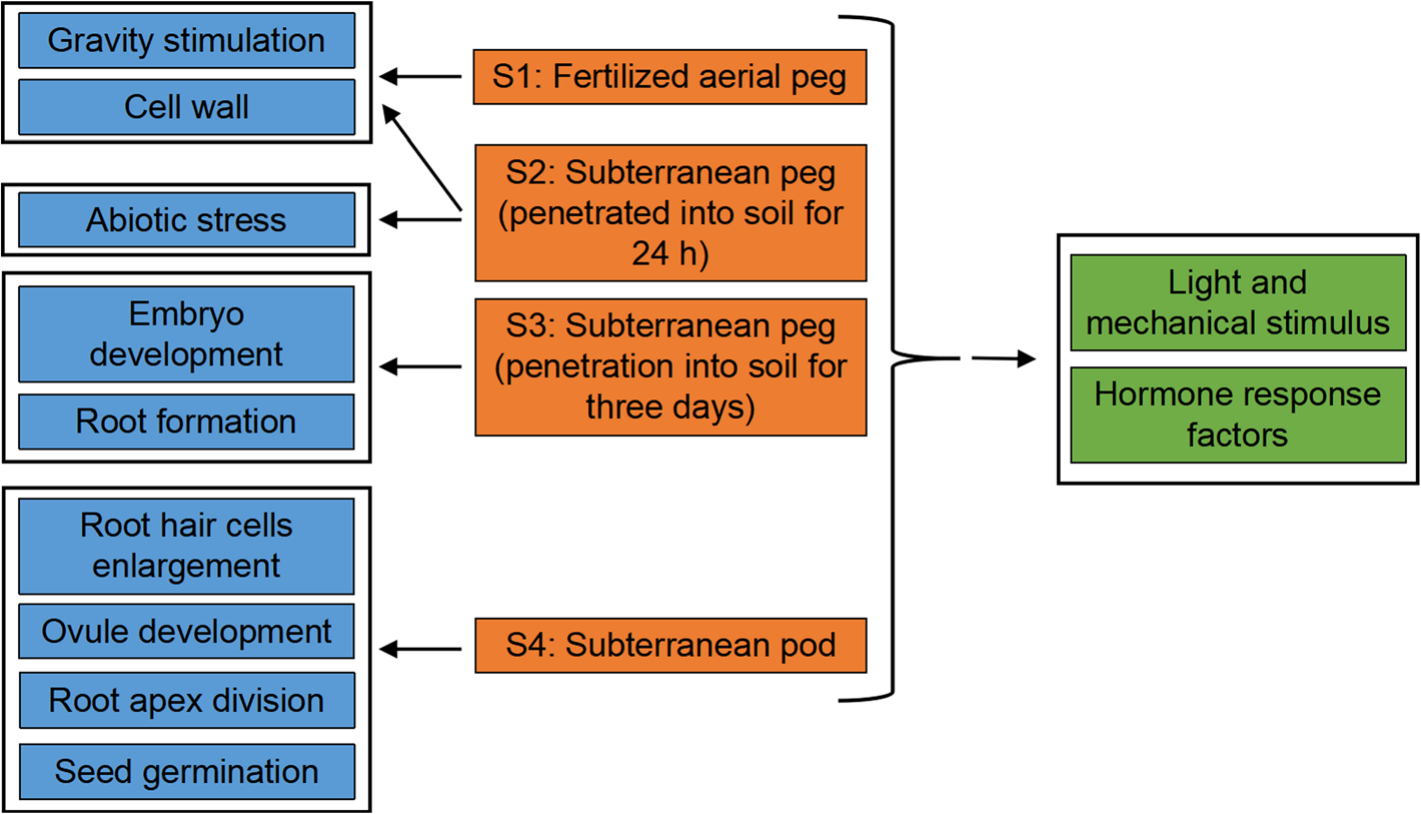
Identified proteins and their suggested function during peanut peg development. The specific splicing isoforms at S1 and S2 stages are related to gravity stimulation and cell wall. Abiotic stress (cold, salt) is involved with specific isoforms at S2 stage. The S3 stage mainly involves embryo development and root formation. The specific isoforms in S4 pegs participate in root hair cells enlargement, ovule development, root apex division, and seed germination. Light and mechanical stimulus, and hormone response factors involve the specific isoforms at all four stages in peanut pegs

In summary, we presented a comprehensive transcriptome profiling of AS isoforms during peanut peg and early pod development. Multiple genes are related to gravity stimulation, light and mechanical response, hormone mediated signaling pathways, and calcium-dependent proteins undergoing AS express development-specific spliced isoforms or exhibit an obvious isoform switch (Fig. 8). The identification of development-specific AS isoforms in subterranean pegs and pods provides valuable sources to further understand post-transcriptional regulatory mechanisms of AS in the generation of pegs and formation of subterranean pods.

## Methods

### Plant material

**Fig. 9:**
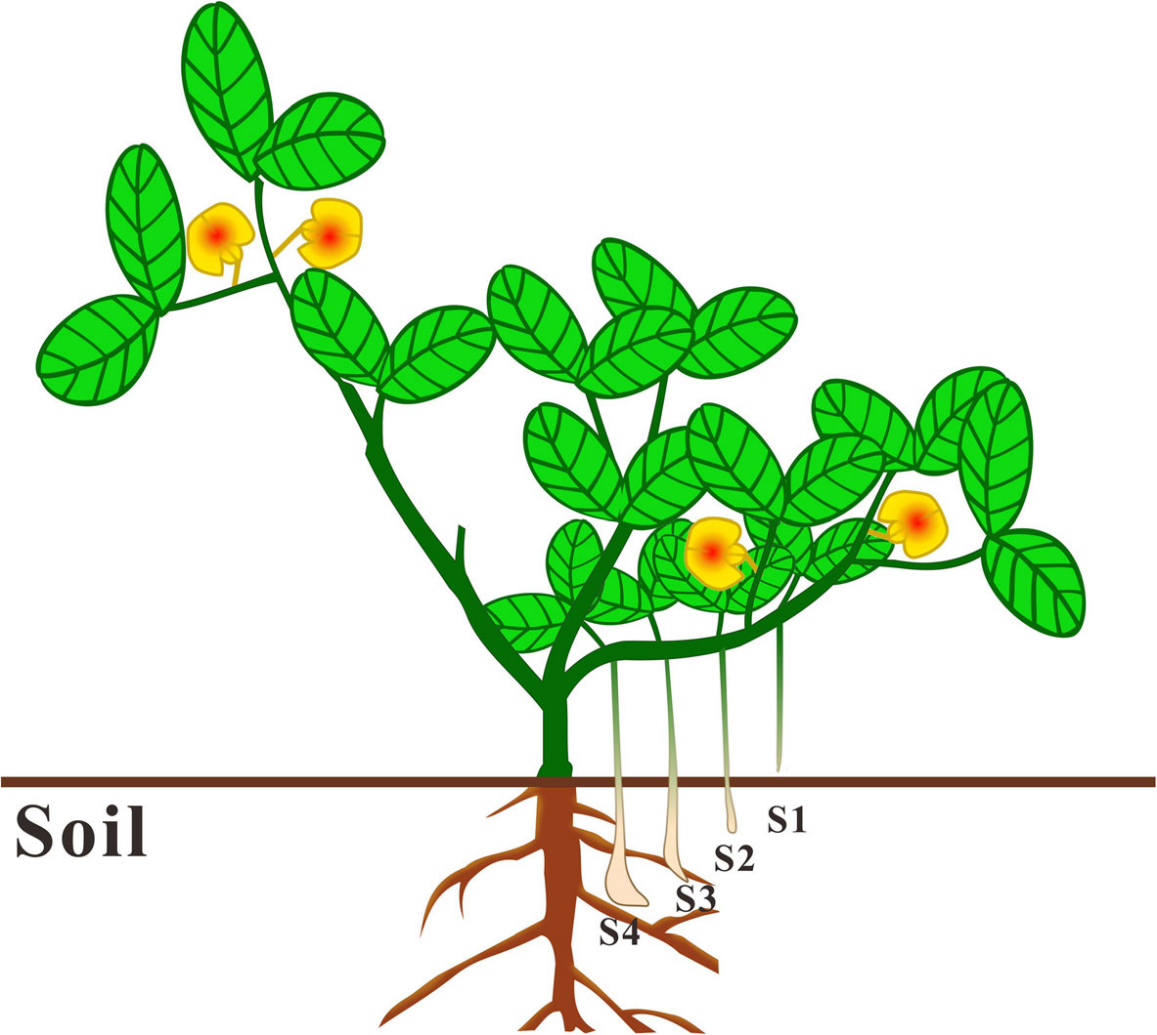
Pictorial representation of sample collection stages for AS analysis. S1: aerial peg tips; S2: subterranean peg tips; S3: subterranean unswollen peg tips; S4: ubterranean swelling pod

Peanut cultivar HY9306 were grown in the glasshouse (25–30 °C) from March to August of the year 2018 at Laixi, Shandong province of China. Peanut seeds were presoaked in deionized water (50 °C) for 10 min, and then incubated overnight at room temperature. Imbibed seeds were germinated in a germination machine (Model CB-A323B, Connie, Guangdong, China) with 100% relative humidity for 5 days in dark. During germination, the temperature was maintained at 25 °C and the seeds were automatically auto-rinsed with fresh deionized water every 10 min. After 10 days, the germinated seeds were planted in vermiculite with one seed per plot. All tissues were collected according to the previous methods described [59]. In brief, root tissues were collected 10 d post emergence. Leaf tissues were sampled seedling leaves 10 d post emergence, main stem leaves, and lateral (n + 1) leaves. Shoot tissues were collected vegetative from main stem and reproductive tips from first lateral (n + 1). Flowers were pooled perianth, gynoecium, and androecium. Peanut pods were sampled aerial peg tips (S1), subterranean peg tips (24 h) (S2), subterranean unswollen peg tips (S3), and subterranean swelling pod (S4) (Fig. 9). All tissues were collected at 14:00 h except for flower samples harvesting at 8:30 h. Each tissue was sampled from ten individual plants. All harvested samples were immediately frozen in liquid nitrogen for 15 min and then stored at − 80 °C.

### RNA isolation

Total RNA was extracted from each tissue sample using GeneJET Plant RNA Purification Kit (Thermo Fisher Scientific, USA). RNA quality was checked by a NanoDrop 2000 instrument (Thermo Fisher Scientific, USA). The RNA integrity was evaluated by an Agilent 2100 Bioanalyzer instrument (Agilent Technologies, USA) and RNA with integrity number above 8.5 was used for subsequent analysis.

### PacBio library construction and sequencing

Qualified RNA from each tissue was equally pooled and used for single-molecule real-time (SMRT) Bell library construction. The SMRTBell libraries were prepared according to the recommended protocols by Pacific Biosciences with following modifications. One microgram total RNA was inputted to synthesize cDNA using SMARTer PCR cDNA Synthesis Kit (Clontech, Japan). PCR amplification was conducted using KAPA HiFi PCR Kits (KAPA biosystems, USA). The PCR products were inputted to BluePippin Size Selection system (Sage science, USA) for size selection and the products with length of 0.5–6 kbp were selected. Then the size selected products were used for SMRTbell Template preparation using SMRTBell Template Prep Kit following the recommend protocols. After SMRTBell libraries were prepared, thirty nanogram of each template were inputted to a PacBio Sequel platform (KeGene Tech, Shandong) to complete sequencing using P6-C4 polymerase. A total of eight SMRTBell libraries were sequenced.

### Illumina library construction and sequencing

Illumina sequencing libraries were completed using Illumina TruSeq RNA Library Preparation Kit v2 (Illumina, USA) following the recommended protocols. Briefly, 1 μg of total RNA were used to synthesize cDNA using SMARTer PCR cDNA Synthesis Kit (Clontech, Japan). Then, 300 ng of cDNA were fragmented into length of 250–300 bp using a Covaris E220 Focused-ultrasonicator (Covaris Inc., Woburn, MA, USA). Subsequently, the sheared cDNA was used to construct the sequencing library using Illumina TruSeq RNA Library Preparation Kit v2 using with 10 cycles of PCR. The libraries were sequenced on an Illumina Hiseq X Ten instrument with paired-end 150 bp strategy by KeGene Science & Technology Co. Ltd. (Shandong, China).

### PacBio Iso-Seq data analysis

The raw polymerase reads obtained were processed using the IsoSeq v3 software (https://github.com/PacificBiosciences/IsoSeq). After removing adaptors and low-quality reads, the clean subreads were processed to get circular consensus sequence (CCS). According to whether presence of 3′, 5′ primers, and poly A tail simultaneously or not, the CCS sequences were classified into full-length non-chimeric (FLNC) reads and non full-length (nFL) reads. Subsequently, the FLNC reads were inputted to cluster and generate final polished FLNC sequences. The polished FLNC sequences were mapped to *A. hypogaea* reference genome [60] using GMAP v2019-12-01 software [61] with parameters setting: --max_introlength-middle = 20,000 --no-chimeras -n 0 --split-large-introns --cross-species. The genome alignments were collapsed to obtain unique transcript loci using cDNA_Cupcake v11.0.0 software (https://github.com/Magdoll/cDNA_Cupcake) with parameter setting: -c 0.8 -i 0.7. Subsequently, the unique transcripts obtained were processed to reconstruct coding genome using Cogent v6.0.0 software (https://github.com/Magdoll/Cogent).

### Gene annotation

To obtain the function of each transcript, the ORFs of each transcript were predicted using TransDecoder v5.5.0 software (https://github.com/TransDecoder/TransDecoder) with a minimum amino acid length of 100. For each transcript, the longest ORF was defined as reprehensive and selected for functional annotation. The protein sequences of reprehensive ORFs were searched against NCBI NR and Swiss-Prot databases using using NCBI BLASTp (BLAST v2.2.28+) software with parameters setting: -evalue 1e-5 -max_target_seqs 1 -outfmt 6. To identify functional protein domain, the protein sequences were searched against Pfam v32.0 database using PfamScan program [62]. GO functions of each transcript were obtained by Blast2GO v5.2 software. KEGG functions for each transcript were annotated against *Arabidopsis thaliana* proteins by online tool of KAAS using BBH method [63].

### Alternative splicing events

To identify AS events, the AStalavista v4.0.1 software [64] was executed using the collapsed gff3 file based on FLNC sequences. Five major AS event types, including intron retention (IR, code: 1^2-,0), exon skipping (ES, code: 1–2^,0), alternative acceptor site (AA, code: 1-,2-), alternative donor site (AD, code: 1^,2^), and mutually exclusive exons (MX, code: 1–2^,3–4^), were identified according to the output files.

### Poly(a) analysis

To identify poly(A) tails, the SMRT analysis pipeline of Pacific Bioscience was employed using the FLNC sequences. The poly (A) sites were determined when there were more than eight A bases and less than two non-A bases in 30 bases for all FLNC sequences. The motif peaks on the flanking of poly (A) sites were screened using online MEME tool.

### Novel transcripts and long intergenic noncoding RNAs identification

The FLNC sequences which were not aligned to the known gene models of peanut reference genome were identified as novel transcripts. Novel transcripts were employed to identify long intergenic noncoding RNAs (lincRNAs). The CPAT v1.2.4 software [65] and PLEK v1.2 software [66] were used to identify lincRNAs from novel transcripts. The lincRNA candidates were compared with previous constructed datasets [67].

### Illumina RNA-seq data analysis

Illumina RNA-seq data were employed to help in detecting splice junctions (SJs). Three tools, TopHat v2.1.1 [68], MapSplice v2.2.1 [69], and STAR v2.7.3a [70], were used for genome mapping. The SJs were retained when there were at least two software identified and at least five RNA-seq reads supported. The expression level of each transcript was estimated by Cufflinks v2.2.1 software [71] with parameters setting: -multi-read-correct -frag-bias-correct. Tissue specificity was determined by calculating Jensen-Shannon (JS) divergence score of each transcript [72].

## Supplementary information

**Additional file 1: Fig. S1.** Characterization of peanut transcriptome data. (a) Pipeline used for reconstruction of FL transcript loci from Iso-Seq. (b) The percentage of multiply and uniquely mapped reads. (c) Distribution of lincRNA length. (d) Distribution of exon number in all lincRNAs.

**Additional file 2: Fig. S2**. Chromosomal landscape of isoforms in reference annotation and PacBio data. Data type that each track represents is shown in left corner. The inner lines show loci for fusion genes. For all the tracks, each chromosome was divided into 1 Mb bins sliding 200 kb.

**Additional file 3: Table S1**. Statistics of transcriptome data generated by Iso-seq.

**Additional file 4: Table S2**. Identification of AS events from all transcriptome.

**Additional file 5: Table S3**. GO enrichment analysis of isoforms in cluster 1 and 2.

**Additional file 6: Table S4**. GO enrichment analysis of specific isoforms at each stage.

**Additional file 7: Table S5**. Specific transcripts in each peg tissue.

## Data Availability

The datasets used and analyzed in the current study are available from the corresponding author on reasonable request. Sequences have been deposited in NCBI Sequence Read Archive under project PRJNA643877.

## Abbreviations

AS: Alternative splicing
FL: Full length
FLNC: Full-length non-chimeric reads
nFL: Non full-length
ORF: Open read frame
UTRs: Untranslated regions
lincRNAs: Long intergenic noncoding RNAs
IR: Intron retention
AA: Alternative adaptor
AD: Alternative donor
ES: Exon skipping
MX: Mutually exclusive exon
SC: Single copy (region)
SMRT: Single-molecule real-time
SJs: Splice junctions
JS: Jensen-Shannon
