# Supplementary figures and images for: Alternative splicing profiling provides insights into the molecular mechanisms of peanut peg development
Source: BMC Plant Biol. 2020 Oct 23;20:488. doi: 10.1186/s12870-020-02702-y (PMC7585205; doi:10.1186/s12870-020-02702-y)

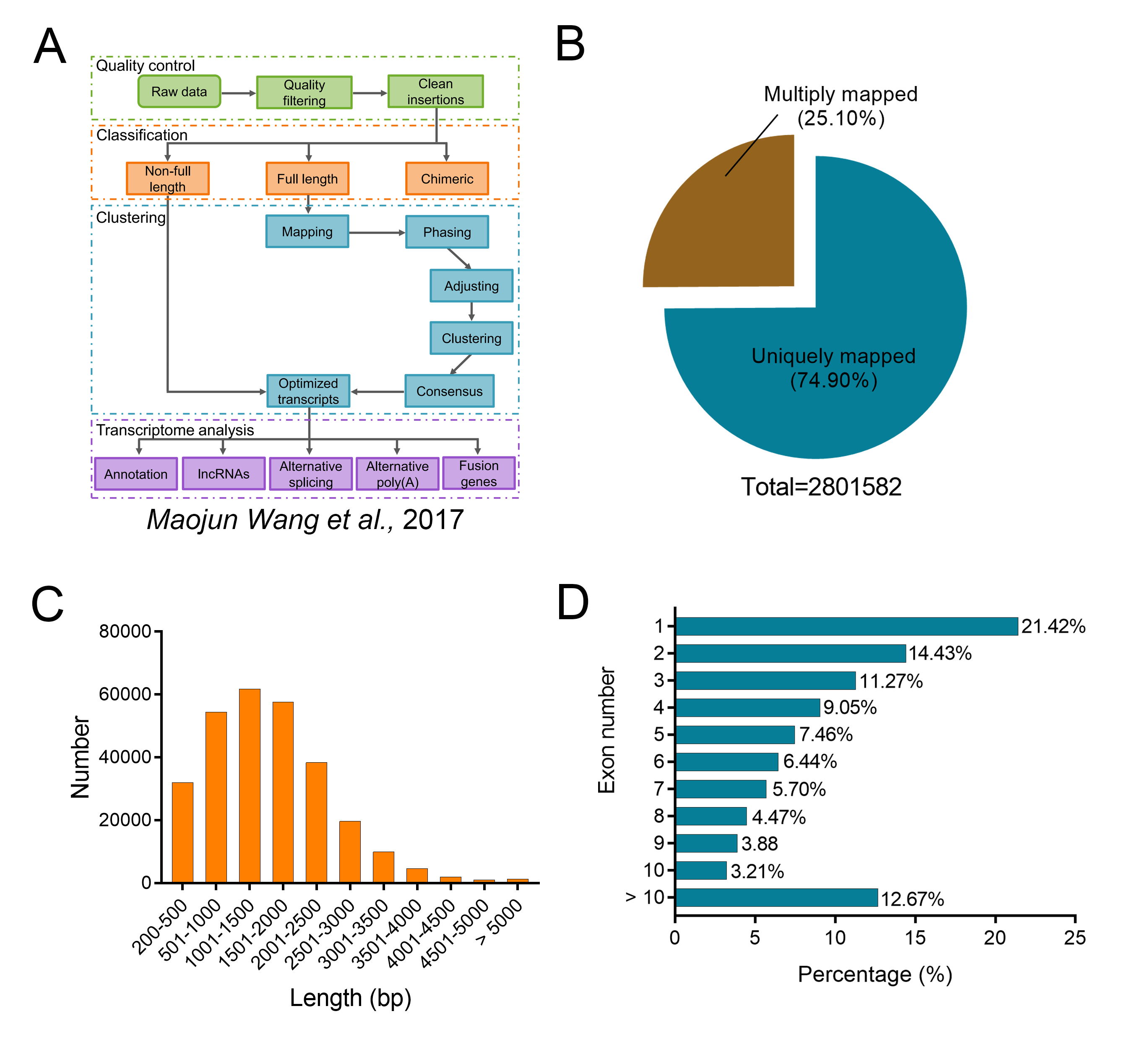

Supplement: Supplementary file 1 — Additional file 1: Fig. S1. Characterization of peanut transcriptome data. (a) Pipeline used for reconstruction of FL transcript loci from Iso-Seq. (b) The percentage of multiply and uniquely mapped reads. (c) Distribution of lincRNA length. (d) Distribution of exon number in all lincRNAs. [file 12870_2020_2702_MOESM1_ESM.tif]

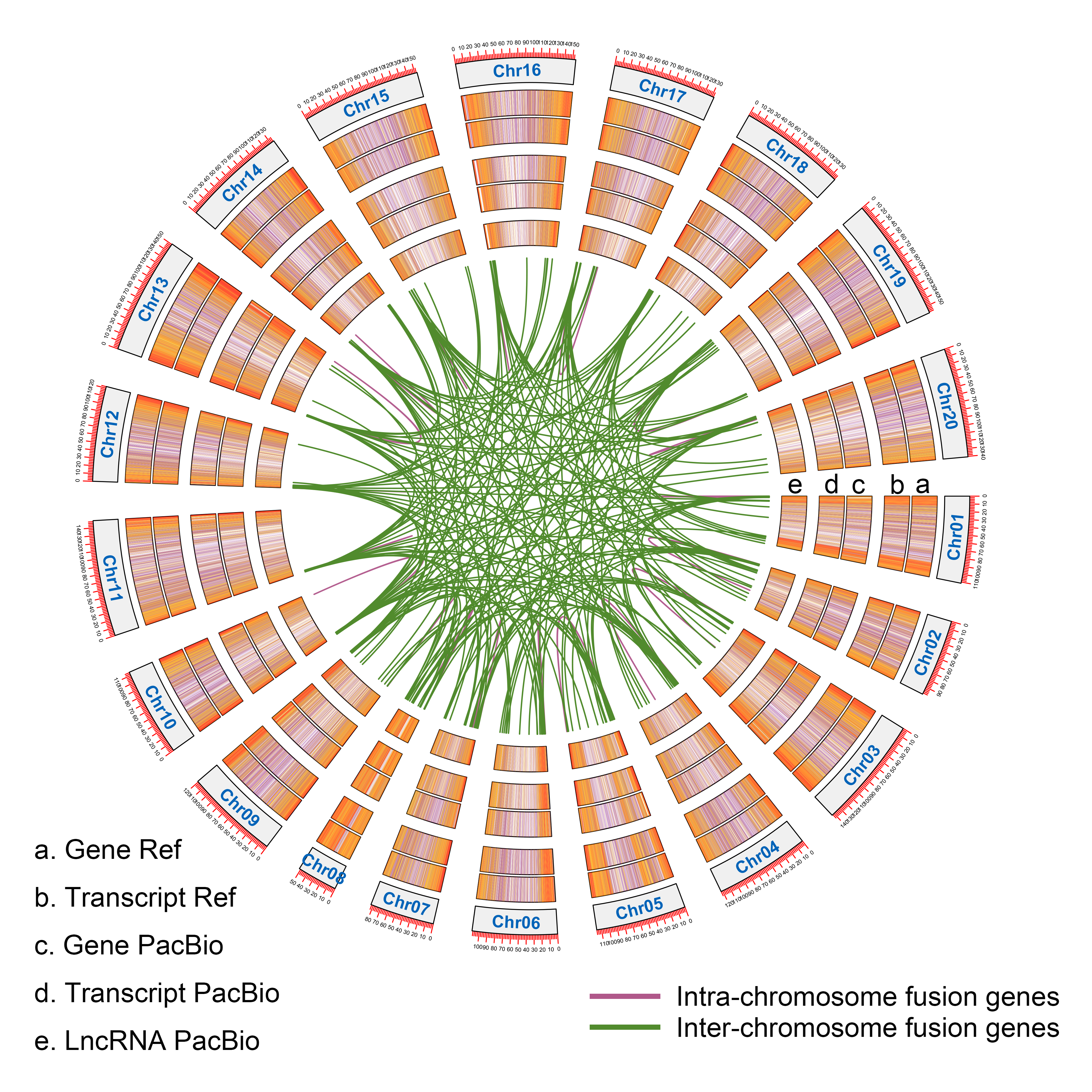

Supplement: Supplementary file 2 — Additional file 2: Fig. S2. Chromosomal landscape of isoforms in reference annotation and PacBio data. Data type that each track represents is shown in left corner. The inner lines show loci for fusion genes. For all the tracks, each chromosome was divided into 1 Mb bins sliding 200 kb. [file 12870_2020_2702_MOESM2_ESM.tif]
